# An innovative approach based on real-world big data mining for calculating the sample size of the reference interval established using transformed parametric and non-parametric methods

**DOI:** 10.1186/s12874-022-01751-1

**Published:** 2022-10-20

**Authors:** Chaochao Ma, Li’an Hou, Yutong Zou, Xiaoli Ma, Danchen Wang, Yingying Hu, Ailing Song, Xinqi Cheng, Ling Qiu

**Affiliations:** 1 Department of Laboratory Medicine, Peking Union Medical College, Peking Union Medical College Hospital, Chinese Academy of Medical Science, 100730 Beijing, PR China; 2 State Key Laboratory of Complex Severe and Rare Diseases, Peking Union Medical College, Peking Union Medical College Hospital, Chinese Academy of Medical Science, 100730 Beijing, PR China

**Keywords:** Data mining, Reference interval, Sample size

## Abstract

**Background:**

Currently, the direct method is the main approach for establishment of reference interval (RI). However, only a handful of studies have described the effects of sample size on establishment of RI and estimation of sample size. We describe a novel approach for estimation of the sample size when establishing RIs using the transformed parametric and non-parametric methods.

**Methods:**

A total of 3,697 healthy participants were enrolled in this study. We adopted a two-layer nested loop sample size estimation method to determine the effects of sample size on RI, using thyroid-related hormone as an example. The sample size was selected as the calculation result when the width of the confidence interval (CI) of the upper and lower limit of the RI were both stably < 0.2 times the width of RI. Then, we calculated the sample size for establishing RIs via transformed parametric and non-parametric methods for thyroid-related hormones.

**Results:**

Sample sizes for thyroid stimulating hormone (TSH), as required by parametric and non-parametric methods to establish RIs were 239 and 850, respectively. Sample sizes required by the transformed parametric method for free triiodothyronine (FT3), free thyroxine (FT4), total triiodothyronine (TT3) and total thyroxine (TT4) were all less than 120, while those required by the non-parametric method were more than 120.

**Conclusion:**

We describe a novel approach for estimating sample sizes for establishment of RI. A corresponding open-source code has been developed and is available for applications. The established method is suitable for most analytes, with evidence based on thyroid-related hormones indicating that different sample sizes are required to establish RIs using different methods for analytes with different variations.

**Supplementary Information:**

The online version contains supplementary material available at 10.1186/s12874-022-01751-1.

## Introduction

Reference intervals (RI) play an important role in clinical practice, mainly in assessment of patients’ conditions during disease diagnosis. Sample size selection during RI establishment is important to ensure its stability [1, 2]. A limited number of studies have described the effects and methods for establishment of sample sizes for RI determination. In 2010, EP-28A3c [3] suggested that the sample size for establishment of RI should not be less than 120, although this was mainly based on a simple non-parametric approach in which a minimum of 120 samples are required for calculating a 90% confidence interval (CI) of the reference limit. Although such recommendations are convenient and feasible, 120 samples are far from enough for establishment of RI, especially for some analytes with large variations [1]. Studies have reported on the effects of sample sizes on establishment of RIs [1, 4, 5], while others have shown that reference limits should be assessed against their CIs, which should be < 0.2-fold the width of RIs [6, 7].

Biological parameters fluctuate from analyte to analyte, therefore, sample sizes for establishment of RI should vary for different clinical laboratory tests. The significance of sample sizes for establishing RIs has been reported [8–12]. However, very few methods have been developed for estimating sample sizes for establishment of RIs [9, 13]. Based on the report by Henny et al. [6], we aimed at developing a method for estimating sample sizes for establishment of RIs. Moreover, we developed a code for this method and used it to calculate the sample size required to establish RIs for thyroid-related hormones based on both parametric and non-parametric methods. This method can be used to calculate and evaluate sample sizes when establishing RIs using the direct method in different clinical laboratories.

## Materials and methods

### Study participants and selection criteria

A total of 3,697 healthy participants were enrolled from among those attending Peking Union Medical College Hospital for routine check-ups between 1st January 2014 and 29th December 2018. Information on participants’medical history, including their symptoms and past history among others were confirmed via a review of electronic medical records. The inclusion criteria were individuals: (i) Without a history of acute or chronic diseases, including respiratory, circulatory, digestive, urinary, and autoimmune diseases, as well as acute and chronic infections, metabolic and nutritional diseases, blood system diseases, endocrine diseases, and cancers; (ii) With 18.5 kg/m2 ≤ BMI < 24 kg/m2; (iii) Whose systolic and diastolic blood pressures were < 140 and < 90 mmHg, respectively; (iv) With negative anti-thyroglobulin antibodies (TG-Ab) and anti-thyroid peroxidase autoantibodies (TPO-Ab); (v) Whose thyroid ultrasound was normal; (vi) Who were aged ≥ 18 years and (vii) Whose routine biochemical levels, such as ALT, Cr and Glu, were within the RI or less than the medical decision level.

Pregnant women were excluded from this study, according to the hospital physical examination information system. Furthermore, the sex ratio of the subjects in the study was adjusted to 1:1.

### Analytical performance of analytes

Levels of thyroid related hormones (thyroid stimulating hormone (TSH), free triiodothyronine (FT3), free thyroxine (FT4), total thyroxine (TT4) and triiodothyronine (TT3)) were measured on the ADVIA Centaur XP chemiluminescence immunoassay analyzer (Siemens Healthineers, Erlangen, Germany), using the reagents and calibrators supplied by the systems’ manufacturer. Levels of TG-Abs and TPO-Abs were measured using Roche kits (Cobas e601; Roche Diagnostics), as instructed by the manufacturer. A summary of methods, units and types of samples used for analyses are presented in Supplemental Table 1.

### Data collection

Sample collection and processing procedures were as previously described [1, 14]. Briefly, each participant’s information, including demographics, clinical laboratory data and clinical related information were downloaded from the Laboratory Information System and Hospital physical examination information system. To validate our method, we downloaded the dataset of healthy individuals’ adrenal glands from the GTEx database (https://gtexportal.org/home/). We selected four genes (NAP1L4, OTUD5, UBE2I and DEDD) and used a method established in our study to calculate the sample size.

### Quality control

We established an internal QC data set, with all internal QC data collected during the study period reviewed to ensure accuracy and reliability. In addition, our laboratory is certified by ISO15189 and the COLLEGE OF AMERICAN PATHOLOGISTS (CAP), while the instruments used are regularly maintained as required. The assay platform for the analyte did not change during the time period covered by the data. We double-checked the results from each statistical analysis software or programming to ensure accuracy. Moreover, we invited professionals to review and verify the design, method and code adopted in the study to affirm the correctness of the research plan and analytical method.

### Statistical analysis

All data were recorded in Excel 2016 (Microsoft, Redmond, WA, USA) and analyzed using packages implemented in R language (version 4.0.5), as well as SPSS 25.0 Software (IBM Inc., Armonk, NY, USA) and Medcalc Statistical Software 18.116.6 (Mariakerke, Belgium). Data distribution are presented using histograms of frequency distribution. The kolmogorov-Smirnova test was used to determine data normality. The skewed continuous variables were described as median (25th, 75th centile).The transformed parametric and non-parametric methods were separately used to calculate the 95% RI. Since the robust method is suitable for establishing RI with small samples, we did not calculate the sample size of the method. The Tukey’s method was performed to identify outliers. If data were not normally distributed, the Box-Cox algorithm was used to improve normality. Since we aimed at comparing parametric and non-parametric methods, to objectively compare the two methods, the bootstrap method rather than the calculation formula was used to calculate the 90% CI.

### Sample size calculation method

**Fig. 1:**
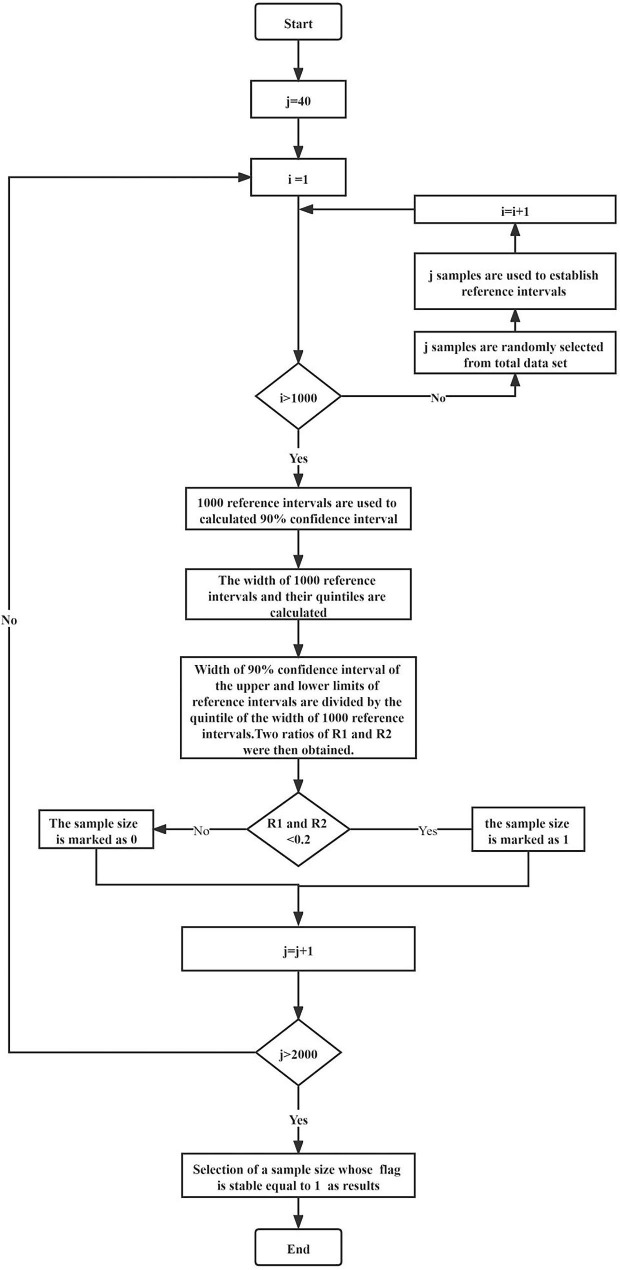
The flow chart of sample size calculation method

The sample size calculation method adopts a two-layer nested loop approach, based on the following steps (Fig. 1):

1. The first layer cycle changed the sample size from 40 to 2000 (the pre-experimental results indicated that all five thyroid-related hormones achieved convergence before 2000), the iteration step of the sample size is fixed at 1.
2. The second layer of the loop is sampling with replacement. Under the sample size set in the first layer, 1000 times of sampling were performed. Thereafter, the upper and lower limits of RI were calculated according to the transformed parametric and non-parametric methods, respectively. After completion of 1000 times of return sampling, corresponding 90% CI for upper and lower RI were calculated.
3. The widths of 1000 RI and their quintiles were calculated.
4. Under a set sample size in the first layer cycle, the width of 90% CI of the upper and lower limits of RI were divided by the quintile of the width of 1000 RI, respectively. Two ratios of R1 and R2 were then obtained.
5. If both R1 and R2 were less than 0.2, the sample size was marked as 1; otherwise, it was marked as 0. Label variable of the sample size was obtained in this step.
6. Taking 10 as moving window width, the sample label variable was summed by moving summation method, to obtain summation variables.
7. Taking 10 as the moving window width, the moving median method was used to calculate the moving median of the summation variable, and moving median variables obtained.
8. When the moving median was equal to 10, for the first time, the corresponding sample size was the estimated minimum sample size required to establish RI for the analyte.

#### Note:

Steps 6, 7 and 8 are aimed at reducing the influence of fluctuation of the width of reference and confidence intervals with increasing sample size on result of sample size calculation. That is, select the sample size whose flag is stably equal to 1.

These calculations were performed using a code written in R language (Provided in Supplemental materials) .

## Results

### Baseline information of enrolled subjects

**Fig. 2:**
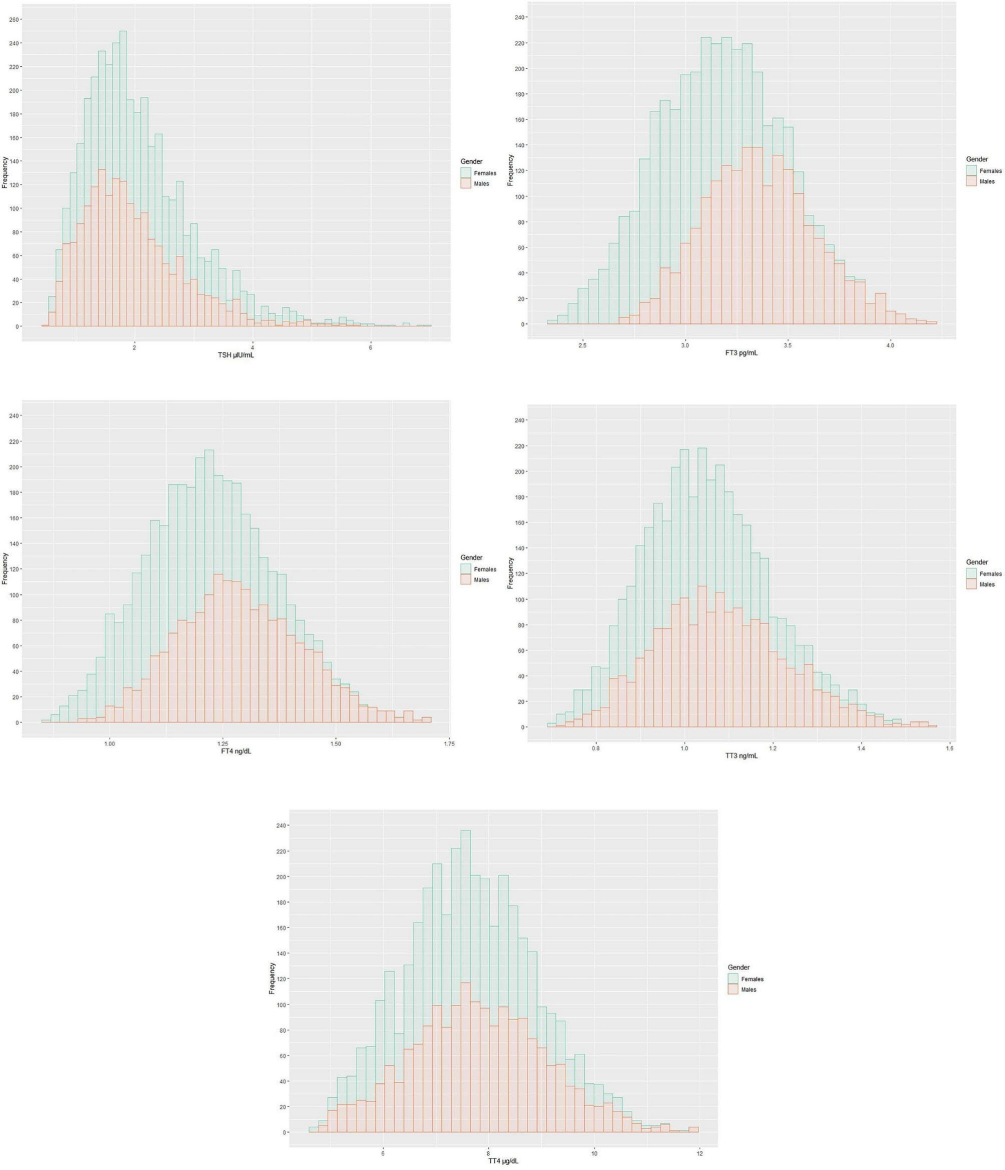
Distribution of thyroid-related hormones in the study. Green represents male frequency distribution, red represents female frequency distribution, and the whole contour represents the overall distribution

The ratio of males to females in this study was 1:1. Their median age was 33 years, with minimum and maximum ages of 18 and 84 years, respectively. The distributions of TPO-Ab and TG-Ab were 11.31 (10.00, 14.20) IU/L and 11.06 (8.54, 14.10) IU/L, respectively. Thyroid-related hormone distributions were as shown in Fig. 2. In this study, TSH exhibited a right skewed distribution, whereas FT3, FT4, TT3 and TT4 exhibited an approximate normal-distribution.

### Effects of sample size on reference intervals of thyroid-related hormones

**Fig. 3:**
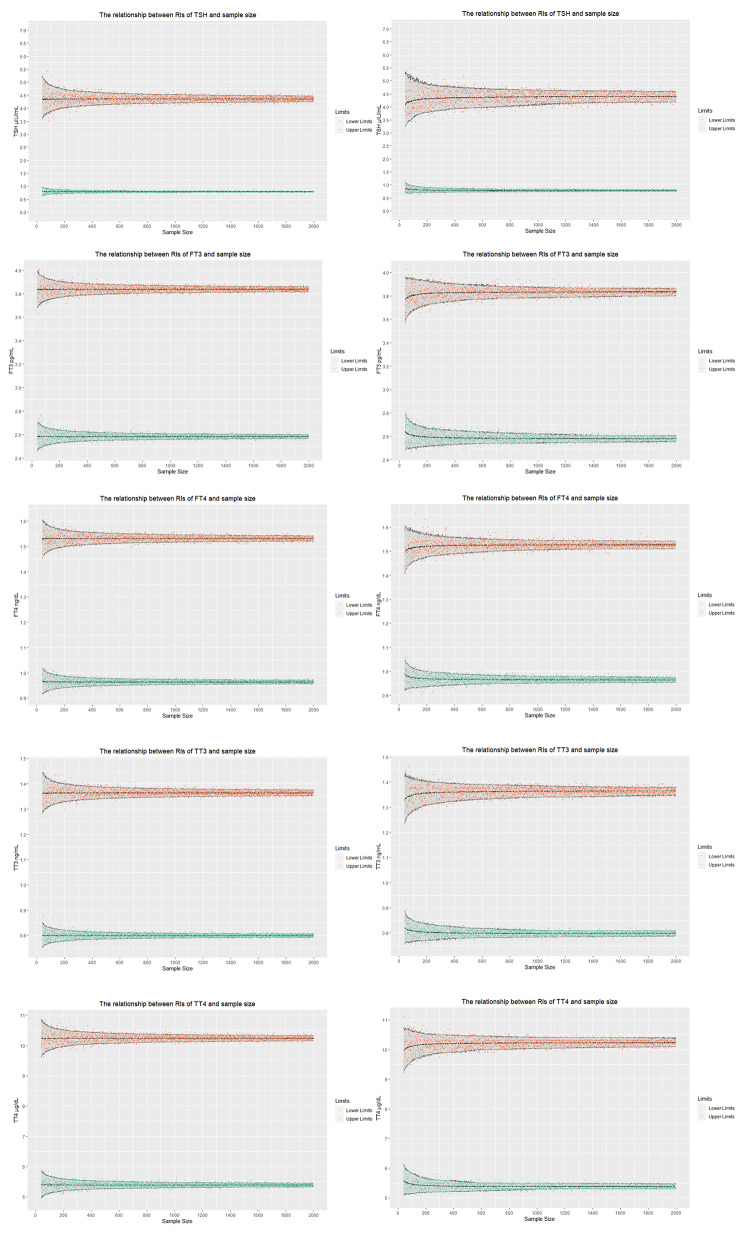
Relationship between reference intervals of thyroid hormones and sample size. Briefly, the orange and cyan scatter points represent results of one in 1000 random samples, under different sample sizes. That is, the upper limit and lower limit of the RIs of the thyroid-related hormones under various sample sizes. The area enclosed by the grey band and black dotted lines is the 90% CI of the upper and lower limits of the RIs for thyroid-related hormones in different sample sizes. The black dotted line, in the center of the confidence band, represents the mean of the upper or lower limits of the RIs for 1000 samples under the same sample size. The methods for establishing RIs include transformed parametric and non-parametric methods in the figure on the left and the figure on the right, respectively

The relationship between upper and lower limits of RI for TSH, FT3, FT4, TT3 and TT4 with sample size are shown in Fig. 3. CI of limits of RIs tended to shrink with increasing sample size, and the narrowing trend of transformed parametric method was faster than that of the non-parametric method. Visually, the CI of non-parametric method is slightly wider than that of the transformed parametric method for the same sample size. For the five thyroid-related hormones, the upper limit of RI had greater variations than the lower limit.

### Sample size for establishment of RIs for thyroid-related hormones

We used our proposed method to calculate the sample size for establishing the RIs (Table 1). The sample size required by the transformed parametric method to establish RI, for the five thyroid-related hormones, was smaller than that required by the non-parametric method. This is consistent with the law shown in Fig. 3, where 90% CI of the RI boundary value of the non-parametric method is wider than that of the transformed parametric method. For TSH, the sample size required by both the transformed parametric and non-parametric methods to establish the RIs is greater than 120. Conversely, the sample sizes required by the transformed parametric method for FT3, FT4, TT3 and TT4, are all less than 120, whereas those required by the non-parametric method are more than 120.

**Table 1:** Sample size of establishment of RIs for thyroid-related hormones

Analytes	Biological variation (%)		Units	Method	Sample Size	Validation	
						**LLW**_**CI**_ **/W**_**RI**_	**ULW**_**CI**_ **/W**_**RI**_
TSH	Between-subject	35.0	µIU/mL	Transformed parametic method	239	0.037	0.194
	Within-subject	21.2		Non-parametric method	850	0.026	0.153
FT3	Between-subject	6.0	pg/mL	Transformed parametic method	75	0.170	0.168
	Within-subject	16.5		Non-parametric method	179	0.149	0.178
FT4	Between-subject	7.7	ng/dL	Transformed parametic method	90	0.118	0.197
	Within-subject	10.7		Non-parametric method	197	0.126	0.189
TT3	Between-subject	9.4	ng/mL	Transformed parametic method	97	0.096	0.227
	Within-subject	12.2		Non-parametric method	192	0.138	0.198
TT4	Between-subject	6.4	µg/dL	Transformed parametic method	80	0.212	0.144
	Within-subject	12.0		Non-parametric method	161	0.120	0.169

Data of biological variation derived from https://biologicalvariation.eu/ (2021.12.24)

LL, lower limits; UL,upper limits; W_CI_, width of confidence interval; WRI, width of reference interval

Note: The bootstrap method was used to calculate 90% confidence intervals for the non-parametric method.

Findings for GTEx datasets are shown in Supplemental Tables 2 and Fig. 1. The sample sizes for establishment of RIs using the transformed parametric method calculated by our method for four gene TPMs were all less than 120, while those of the non-parametric method exceeded 120.

## Discussion

We developed an innovative approach for estimating sample sizes for establishment of RIs. We assessed the CIs for various statistical approaches used as direct methods by calculating indirect RIs and their CIs using various sample sizes. Our sample size calculation method adopts the two-layer nested loop, with the first layer circulating the sample size, while in the second layer loop, CI of the limit value of the RI through repeated sampling is calculated under the sample size set in the first layer loop. The loop stop condition was set according to views of Henny et al. [6]. Furthermore, we did not first select the sample size that meets the cycle stop condition as the calculation result, which was due to fluctuations in widths of CI of RI caused by changes in sample sizes. Instead, we adopted the method of moving sum and median, with a window width of 10, for selection of a sample size that is first stably achieved by cycle stop condition.

Based on the above methods, we selected thyroid-related hormones for analyzing the effects of sample sizes on RI. Next, we calculated the sample sizes of RIs for thyroid-related hormones using transformed parametric and non-parametric methods. The transformed parametric method resulted in faster narrowing of the width of CI of the reference limits of RI, compared to the non-parametric method, consistent with findings from our previous study [1]. Moreover, our findings indicated that sample sizes required to establish RI for thyroid-related hormones, using non-parametric parameters, were all greater than 120. The sample size required to establish the RI for TSH, using the transformed parametric method, was more than 120, although this value was less than 120 for FT3, FT4, TT3 and TT4, using the same method. Previously, we found that the stable sample size for TSH distribution was around 1500, based on visual distributions [1]. However, we did not determine specific sample sizes, and the conclusion that a sample size greater than 120 should be used for establishing RI of TSH was based only on visual observations due to large sample size group spacing. For the right skewed data like TSH, the CI width on the right side of the tail of the data was larger than that on the left. This may explain why it is difficult to estimate the upper limits of RI for analytes with a right skewed distribution. Findings from the GTEx databases exhibited the same patterns, that is, the transformed parametric method caused faster narrowing of the width of CI of the reference limits for RI, compared to the non-parametric method.

The findings in this study, based on the established method, gives a more accurate estimation. For TSH, these results suggest that a sample size greater than 120 should be selected, regardless of the method (transformed parametric or non-parametric) is used. The upper and lower limits of TSH RI obtained by the non-parametric method using 120 samples exhibited large variations. For the other four thyroid-related hormones, we believe that if the transformed parametric method is used, a sample size less than 120 should suffice. In addition, the idea that at least 120 samples should be used for establishing RI was proposed for non-parametric methods in EP28-A3c more than 10 years ago [3]. At the time, the guidelines recommended using the non-parametric method because it was easy to operate and had no requirements on data distribution. Moreover, since non-parametric calculations require at least 120 samples for calculation of 90% CI, there is a widely held consensus that RI requires at least 120 samples. However, it has been more than a decade since these guidelines were published, and in the intervening years, rapid advances in computing power and information technology have generated more sophisticated statistical methods. Therefore, we postulate that a small sample size is enough for establishing the RI for some analytes with small variations, especially when the transformed parametric method is used. In this study, we found that a sample size greater than 120 should be selected regardless of whether the transformed parametric or non-parametric method is used for indices with large variations, like TSH.

Various methxds for calculating sample sizes to establish RIs have been developed, and they involve many procedures [10, 11]. These methods use a number of formulas to calculate the sample size. Compared to these methods, we provide a more understandable approach, a personalized approach, using real-world big data for estimation.

This study, based on real-world big data, has several advantages. First, the method for estimation of sample sizes proposed herein, which is based on variations across analytes and uses real-world data to estimate sample sizes for establishment of RI, is low cost and has strong practicabilities. Second, we developed a corresponding open-source code for the established method, which is convenient for use by other scholars seeking to calculate sample sizes of RIs for different analytes. Last, since the distribution and variations of analytes in special groups, such as the elderly, may differ from those in -non-elderly adults, it is important to calculate the sample size required for establishing RI for the elderly. The method proposed herein can be used in such cases.

This study has some limitations. First, there are no historical datasets available when calculating sample sizes for new assays. Second, only analytes that have been validated and can be used to establish reference intervals using big data can be estimated using this method. This is because if the data distribution obtained through the real-world big data, such as population undergoing physical examination, is different from the real apparent healthy individuals, then, the estimated results are bound to be biased. However, since some previous studies have demonstrated the feasibility of using big data to establish RIs [14–18], and others affirmed the feasibilities of such approaches [19–22], this method is suitable for most analytes. However, this study does not relate to sample size requirements for data mining approaches using Hoffmann, Bhattacharya and DKGL statistical approaches. Studies should aim at calculating sample sizes of common analytes and to elucidate on the relationship between variations of analytes with sample sizes required to establish RIs. Finally, we aimed at establishing a model that can be used to calculate a sample size based on analyte variations. Last but not least, the new approach in this study examined CIs for statistical approaches typically used for direct methods by calculating indirect reference intervals and their CIs at various sample sizes. This is based on the fact that the population (e.g. healthy individuals who present themselves to hospitals for routine check-ups) used to establish the reference interval by the indirect method have a very low probability of disease and the outliers are removed by the robust methods. Therefore, if patient data or a confounding data are used to estimate the sample size based on the method of this study, a wrong result may be obtained.

## Conclusion

This study provides an innovative approach, and an open-source code for estimating a sample size for establishment of RI. The proposed method is applicable for most analytes, with evidence from thyroid-related hormones (taken as an example) revealing that different sample sizes are required by different methods to establish RIs for analytes with variations.

## Electronic supplementary material

Below is the link to the electronic supplementary material.

Supplementary Material 1

## Data Availability

One dataset analysed during the current study are available in the GTEx repository, [https://gtexportal.org/home/] the other dataset used and analysed during the current study available from the corresponding author on reasonable request after approval from the hospital .
