# Supplementary material for: An innovative approach based on real-world big data mining for calculating the sample size of the reference interval established using transformed parametric and non-parametric methods
Source: BMC Med Res Methodol. 2022 Oct 20;22:275. doi: 10.1186/s12874-022-01751-1 (PMC9585851; doi:10.1186/s12874-022-01751-1)
Supplement: Supplementary file 1 — Supplementary Material 1 [file 12874_2022_1751_MOESM1_ESM.docx]

**Supplemental Table 1 The information of analytes**

| **Analytes** | **Units** | **Method** | **Specimen type** |
| --- | --- | --- | --- |
| TSH | μIU/mL | Chemiluminescence | Serum |
| FT3 | pg/mL | Chemiluminescence | Serum |
| FT4 | ng/dL | Chemiluminescence | Serum |
| TT3 | ng/mL | Chemiluminescence | Serum |
| TT4 | μg/dL | Chemiluminescence | Serum |
| TPO-Ab | IU/mL | Electrochemiluminescence | Serum |
| TG-Ab | IU/mL | Electrochemiluminescence | Serum |

**Supplemental Table 2 Sample size of establishment of RIs for gene expression levels quantified with TPM**

| **Gene name** | **Organ** | **Quantization Type** | **Method** | **Sample Size** | **Validation** | |
| --- | --- | --- | --- | --- | --- | --- |
|  |  |  |  |  | **LLW_CI_/W_RI_** | **ULW_CI_/W_RI_** |
| **NAP1L4** | Adrenal gland | TPM | Transformed parametic method | 66 | 0.144 | 0.219 |
|  |  |  | Non-parametric method | 152 | 0.211 | 0.142 |
| **OTUD5** | Adrenal gland | TPM | Transformed parametic method | 89 | 0.183 | 0.137 |
|  |  |  | Non-parametric method | 166 | 0.205 | 0.149 |
| **UBE2I** | Adrenal gland | TPM | Transformed parametic method | 101 | 0.240 | 0.104 |
|  |  |  | Non-parametric method | 195 | 0.207 | 0.051 |
| **DEDD** | Adrenal gland | TPM | Transformed parametic method | 80 | 0.151 | 0.173 |
|  |  |  | Non-parametric method | 169 | 0.227 | 0.054 |

**Data of healthy subjects derived from https://gtexportal.org/home/ (2022.08.04)**

**LL, lower limits; UL,upper limits; W_CI_, width of confidence interval; WRI, width of reference interval.**

**Supplemental Figure 1 The relationship between reference intervals of four gene TPM and sample size**

**
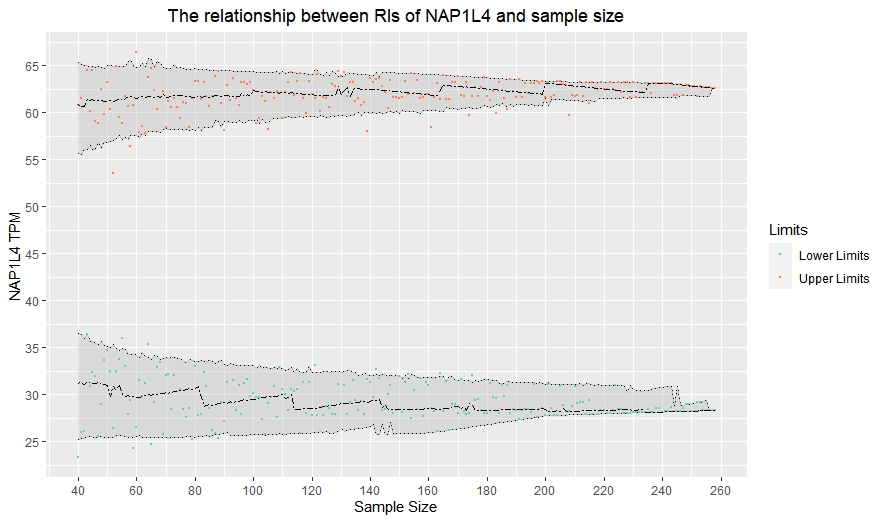

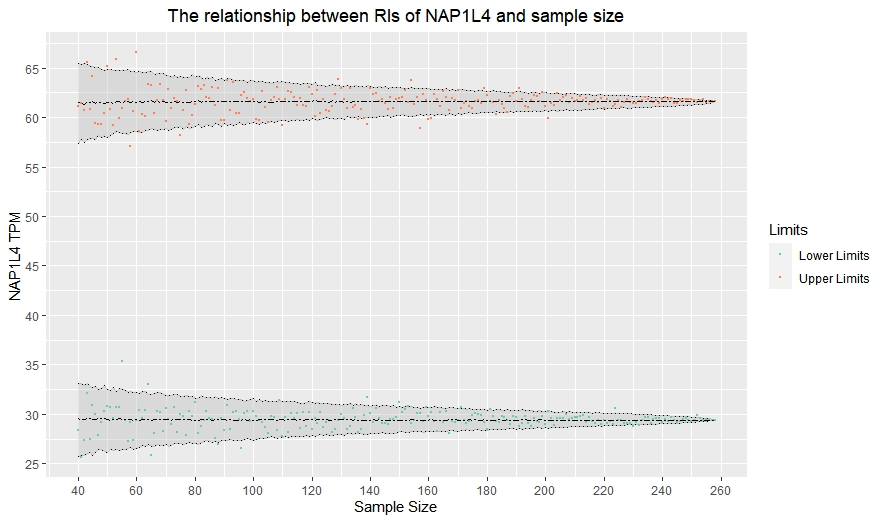
**

**
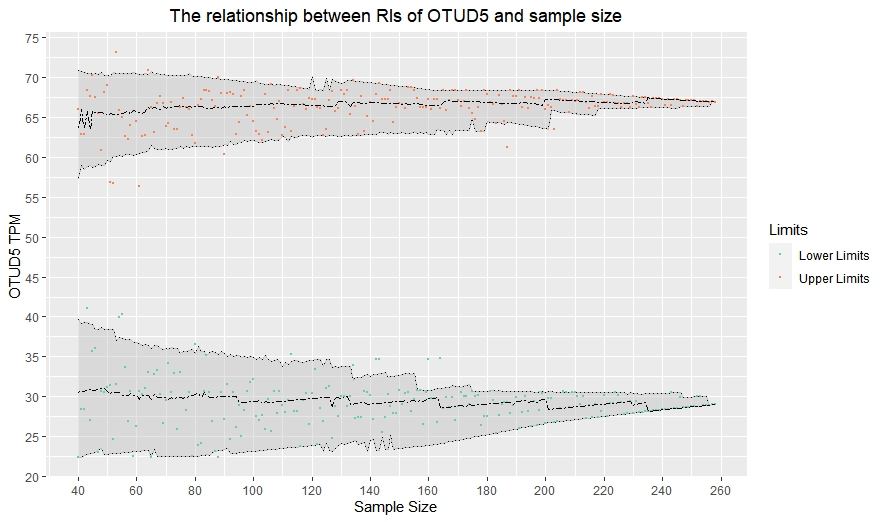

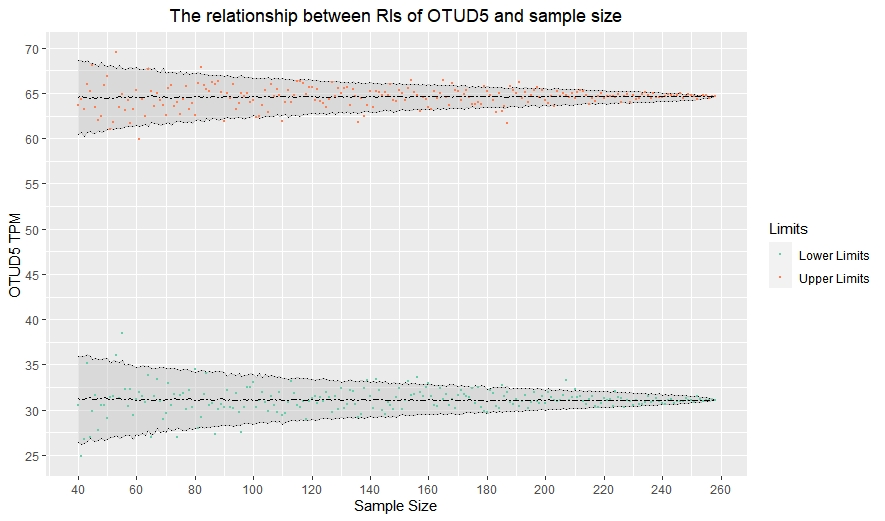
**

**
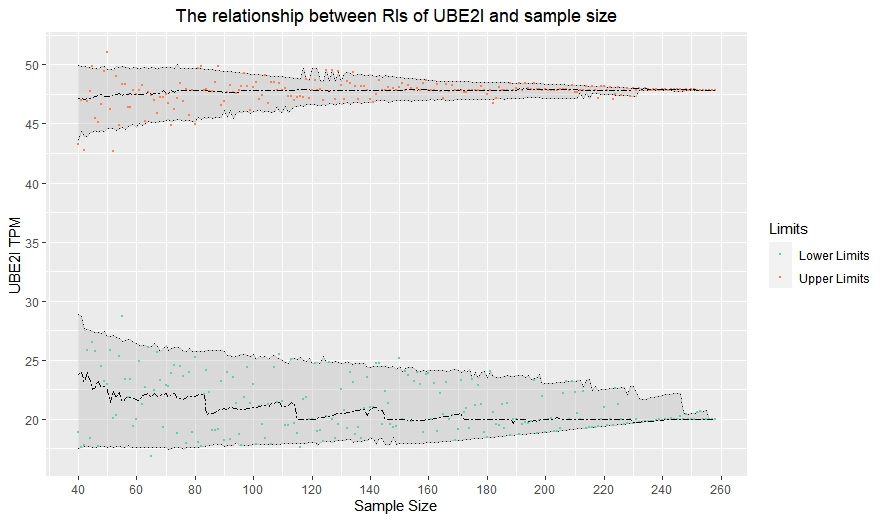

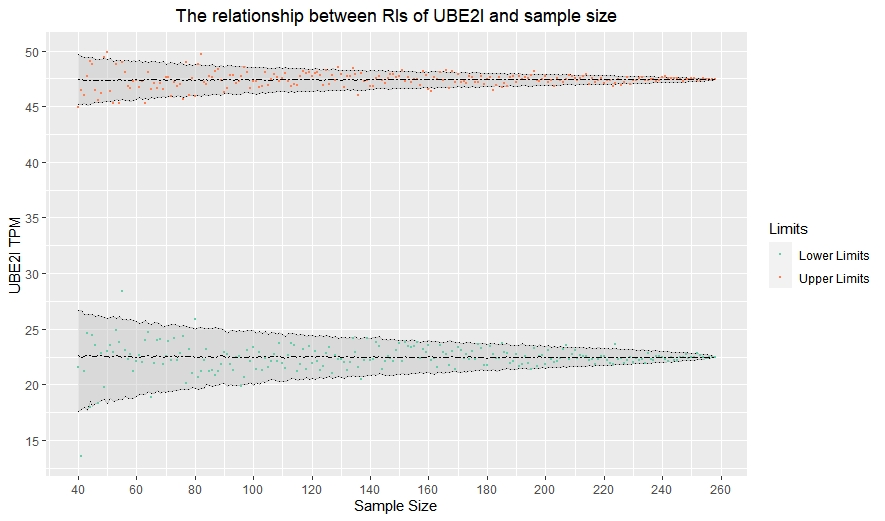
**

**
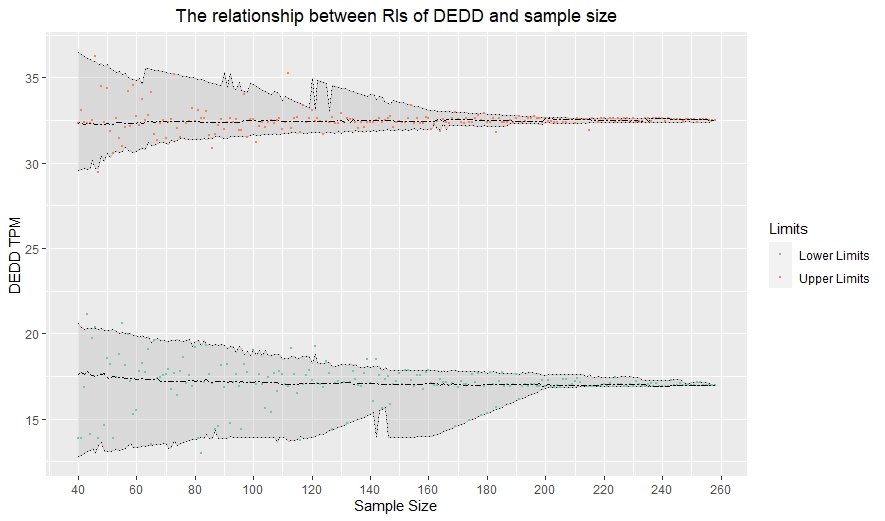

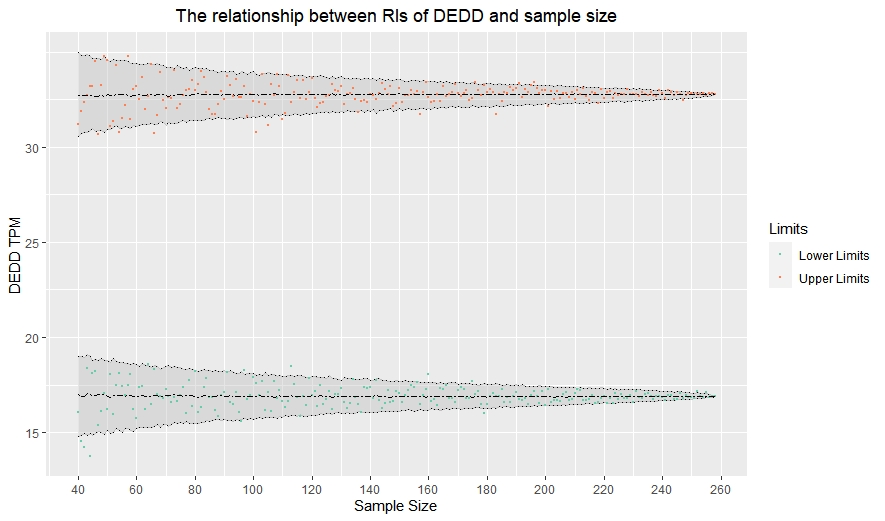
**

The methods of establishing RIs is transformed parametric method and non-parametric method in the figure on the left and the figure right, respectively.
